# Development and validation of a model to predict the risk of frailty in older adults with panvascular disease

**DOI:** 10.3389/fpubh.2025.1631823

**Published:** 2025-11-24

**Authors:** Xia Gao, Cui Xie, Lei Shi, Yuan Li, Rong Hui, Yan Zhang

**Affiliations:** Shaanxi Provincial People’s Hospital, Xi'an, China

**Keywords:** frailty, panvascular disease, older adults, LASSO regression, prediction model validation

## Abstract

**Background:**

This study aimed to identify factors influencing frailty in older adults with panvascular disease and to develop and validate a nomogram-based risk prediction model to support individualized frailty management.

**Methods:**

A total of 1,344 patients aged ≥60 years with panvascular disease were recruited using convenience sampling from March to December 2024. Participants were randomly divided into training and validation sets (7:3). Data included general characteristics, laboratory indices, and scores from the PSQI, ADL, GDS-15, and frailty assessments. Least absolute shrinkage and selection operator (LASSO) regression was used to select predictors, followed by multivariate logistic regression to construct the model. Model performance was evaluated using the area under the receiver operating characteristic curve (AUC), Hosmer–Lemeshow goodness-of-fit test, calibration curves, and decision curve analysis (DCA).

**Results:**

Among 1,344 participants, 366 (27.23%) were frail. LASSO regression identified increasing age, multiple atherosclerotic sites, elevated LDL-C, hypertension history, high HbA1c, and low ADL as significant predictors of frailty. The AUC values for the training and validation sets were 0.78 and 0.77, respectively, indicating good discrimination. The Hosmer–Lemeshow test (*χ^2^* = 4.09, *p* > 0.05) and calibration curve demonstrated strong agreement between predicted and observed outcomes, confirming good model calibration and clinical utility.

**Conclusion:**

The developed nomogram-based model demonstrates strong predictive performance and can objectively estimate frailty risk in older adults with panvascular disease, providing a basis for early screening and targeted prevention strategies.

## 1 Introduction

With the acceleration of global population aging, panvascular disease has emerged as a core public health issue threatening the health and quality of life of the older adults (1, 2). Panvascular disease with systemic atherosclerosis as its pathological foundation, can affect macrovascular, mesovascular, and microvascular systems, with primary target organ damage occurring in the heart, brain, peripheral vascular system, and kidneys (3). The REACH registry study revealed that 25% of coronary artery disease patients present with multi-vascular bed lesions, while this proportion rises to 61.5% among peripheral arterial disease patient (4). Data from the China National Stroke Centre similarly indicate that the prevalence of total vascular disease among ischaemic stroke patients reaches 15.2%, with 8.8% of acute coronary syndrome patients presenting concurrent lower limb and cerebral vascular involvement (5, 6). Although heterogeneity exists in patient inclusion criteria and diagnostic thresholds across studies, these findings collectively indicate high prevalence of panvascular disease in the older adults, with distinct clinical manifestations and management requirements. Significant gaps remain in predicting frailty risk within this population.

Older adults with panvascular disease exhibit distinct characteristics: significantly increased risk of comorbidities (7–9), insidious and atypical symptoms (9, 10), and diffuse vascular lesions (11). However, current frailty risk prediction models for the older adults predominantly focus on single disease entities (12, 13), with scant consideration of panvascular disease frailty. Moreover, some models broadly cover only the older population or those with geriatric comorbidities (14–16). While exhibiting omissions in variable inclusion—for instance, Liu et al.’s (17) frailty prediction model for coronary heart disease patients excluded atherosclerosis risk factors closely linked to pan-vascular diseases, such as glycated hemoglobin or hypertension. Consequently, this study not only incorporates traditional atherosclerosis risk factors like hypertension and glycated hemoglobin (HbAlc), but also innovatively introduces the core indicator of the number of atherosclerotic lesion sites. This provides an evaluative basis for clinically identifying high-risk patients for pan-vascular frailty and formulating personalized comprehensive treatment plans.

The onset and progression of frailty are influenced by multiple factors. The Fried Frailty Scale remains the most widely employed assessment tool, utilizing a simple questionnaire and objective physical tests suitable for both clinical and research settings. Crucially, the Fried frailty phenotype primarily focuses on physiological dimensions of frailty, aligning closely with our geriatric panvascular disease analysis of frailty determinants. In contrast, other mainstream assessment tools exhibit limitations in scenario or target suitability. The Frailty Index (FI) offers comprehensive dimensions but centers on the accumulation of multi-system health deficits and relies on medical data, rendering it more suitable for specific cohorts with comprehensive medical information (18). The Edmonton Frailty Scale (EFS) employs only three rapid screening items, meeting preliminary screening requirements but not comprehensive assessment (19). While the FRAIL scale is convenient, its reliance on subjective symptoms renders it susceptible to accuracy biases from population cognitive interference (20). Notably, the prevalence of the Fried frailty phenotype varies significantly across studies, primarily depending on assessment methods and sample characteristics. A meta-analysis (21) identified age as a core determinant of prevalence—with frailty rates rising from 8.1 to 34.0% across increasing age cohorts. Marital status also showed significant association, with frailty prevalence among unmarried older adults (21.5%) markedly higher than among married older adults (9.0%). This finding further suggests that demographic characteristics such as age and marital status should be incorporated into analyses of frailty determinants among the older population in this study. The sensitivity of the Fried frailty phenotype to these characteristics further validates its suitability as an assessment tool for this research.

## 2 Methods

### 2.1 Study participants

This cross-sectional study used convenience sampling to recruit older adults with panvascular disease at a tertiary hospital in Xi’an, China, between March and December 2024.

The inclusion criteria included: (1) Aged ≥ 60 years; (2) In patients with atherosclerosis in two or more vascular beds confirmed by CT or B-ultrasonography, the main sites include absence of heart, brain, peripheral arteries, etc.; (3) Informed and willing to take part in the study on a voluntary basis.

The exclusion criteria included: (1) History of severe psychiatric symptoms: Refer to DSM-5 (2013) (22), requiring documented diagnosis of major depressive disorder (with hallucinations/delusions), bipolar I disorder (manic episodes), schizophrenia, etc., accompanied by relevant symptom records; (2) Presence of severe systemic disease or advanced malignancy: NYHA Class IV heart failure (23) or Grade 4 disease (including but not limited to severe hepatic failure, non-dialysed Stage 5 chronic kidney disease, etc.) as defined in CTCAE (Version 5.0) (24), or UICC TNM Stage IV cancer (25). Expected survival ≤ 6 months following pathological diagnosis and clinical assessment; (3) Participation in Phase I-IV clinical drug/device trials within the preceding 3 months; (4) Clinical records with incomplete data ≥ 20% missing.

### 2.2 Sample size

This study employed a cross-sectional design and used logistic regression to analyze 20 risk factors for frailty in older adults with panvascular disease. To avoid model overfitting and ensure stable results, the sample size was estimated based on the rule of ‘10 outcome events per predictor variable (EPV = 10)’, requiring 200 frailty events. Given the frailty prevalence of 20–30% in this population (26, 27), a preliminary sample size of 1,000 participants was calculated using the median prevalence of 20%. Considering a 10% non-response rate, the planned sample size was 1,100 participants. In practice, this study included 1,344 participants, meeting the sample requirement. The modeling set and the validation set were randomly divided into a 7:3 ratio, yielding 940 cases for the training set and 404 cases for the validation set.

### 2.3 Data collection

The screening of candidate variables in this study strictly adhered to three core principles: clinical relevance, evidence-based literature, and data accessibility.

#### 2.3.1 Demographic information

General demographic information includes the following questions: (1) Demographic characteristics include age, gender, education level, marital status, smoking history, alcohol consumption, and tumble, hearing and vision, number of atherosclerotic sites, body mass index (BMI). (2) Laboratory indicators: Glycosylated hemoglobin (HbAlc), high-density lipoprotein (HDL-C), triglycerides (TG), and low-density lipoprotein (LDL-C) were obtained from patients’ medical records and laboratory test results from the day of the assessment.

#### 2.3.2 Frailty

Based on Fried’s classic debilitating phenotype assessment system (28). This study assessed five aspects: fatigue (persistent fatigue in the past 4 weeks), endurance deficit (difficulty climbing stairs unaided), impaired mobility (limitation in walking 100 m independently), disease burden (diagnosis of 5 or more of 11 disease categories), and sudden weight loss (≥5% weight loss in 1 year). Each positive item was given a score of 1 and a negative item a score of 0. The total score was 0–5, with 3 and above defined as debilitating and 0–2 as non-debilitating.

#### 2.3.3 Methods for assessing patients’ activities of daily living, sleep quality, and depression symptoms

This study evaluates patient status through three core indicators: The Barthel Index (BI) assesses Activities of Daily Living (ADLs) capability, with a total score ≥100 defining independent ADL performance and <100 indicating dependence; The Pittsburgh Sleep Quality Index (PSQI) developed by Buysse et al. (29) was employed to screen for sleep disorders, with a total score exceeding 7 indicating poor sleep quality. This scale is widely applied in Chinese populations; The Geriatric Depression Scale-15 (GDS-15), a simplified version by Cruice et al. (30) assessed depressive symptoms over the preceding week. A total score ≥8 indicated depressive symptoms, with this scale demonstrating a Cronbach’s *α* coefficient of 0.793 (31). The detailed scoring criteria for all three scales are provided in Appendix A.

### 2.4 Research methods

Prior to the study, the research team explained the study protocol in detail to the relevant departments, made it clear that the research process would not cause any harm to the patients, and gained the departments’ understanding and support. During the study, the researchers first verified the identity of the patients, then distributed self-administered questionnaires, instructed the patients to answer truthfully, and collected medical records on site. A total of 1,344 questionnaires were distributed and collected, and the data were cross-checked and corrected.

### 2.5 Statistical methods

Prior to analysis, the dataset was pre-processed and missing values were imputed using Multiple Imputation by Chained Equations (MICE). According to the data distribution, normal continuous variables were described as mean ± standard deviation (SD) and comparisons between groups were made using the independent samples t-test; non-normal continuous variables were presented as median [*P*_50_ (*P*_25_, *P*_75_)] using the Mann–Whitney *U* test. Categorical variables were presented as percentages (%) using either the chi-squared test or the Fisher exact test, depending on sample size and theoretical frequency.

Variable selection and model coefficient estimation were conducted using the training set, while the validation set was applied for internal validation and evaluation of model performance. This study employed 10-fold cross-validation based on the training set combined with LASSO regression for variable selection, thereby mitigating overfitting. Variables yielding *p* < 0.05 post-selection underwent further multicollinearity testing, with those exhibiting a variance inflation factor (VIF) < 3 ultimately incorporated into the multiple logistic regression model. Regarding model performance evaluation, ROC curves and receiver operating characteristic plots were first generated for risk prediction efficacy analysis. Subsequently, internal validation was performed via 1,000 Bootstrap resampling iterations. The validation set assessment yielded an AUC ≥ 0.7. Calibration curves were employed to verify the consistency between model predictions and actual outcomes, while Decision Curve Analysis (DCA) evaluated the model’s clinical utility. The optimal cutoff value for frailty assessment was determined by maximizing the Youden index (sensitivity + specificity − 1). All statistical tests were two-tailed, with *p* < 0.05 indicating statistical significance. All statistical analyses were performed with SPSS 27.0 and R 4.3.0 software.

## 3 Results

### 3.1 Patient characteristics and baseline comparison

The mean age of the patient group was 71. A total 757 were male (57.66%), 18.53% had a smoking history, and 15.77% had a history of alcohol consumption. Among the panvascular patients, the frailty rates were 27.23% (Table 1).

**Table 1:** Baseline characteristics of the study population.

Variable	Sub-variable	Total *N* = 1,344	Non-frail *N* = 978	Frail *N* = 366	*χ*^2^/*Z*/*t*	*p*-value	
Age (year)	–	71(66, 77)	70(66, 75)	75(68.75, 82)	−8.617	<0.001	
Gender	Male	775(57.66)	548(56.03)	227(62.02)	3.913	0.048	
	Female	569(42.34)	430(43.97)	139(37.98)			
Marital status	Married	1,061(78.90)	785(80.27)	276(75.41)	3.778	0.052	
	Unmarried/divorced/widowed	283(21.06)	193(19.73)	90(24.59)			
Education level	Junior high school and below	748(55.65)	549(56.13)	199(54.37)	7.719	0.021	
	High school or technical secondary school	435(32.37)	300(30.67)	135(36.89)			
	College degree or above	161(11.98)	129(13.20)	32(8.74)			
Smoking	No	1,095(81.47)	809(82.72)	286(78.14)	3.698	0.054	
	Yes	249(18.53)	169(17.28)	80(21.86)			
Alcohol consumption	No	1,132(84.23)	834(85.28)	298(81.42)	2.980	0.084	
	Yes	212(15.77)	144(14.72)	68(18.58)			
Vision	Poor	626(46.58)	490(50.10)	136(37.16)	17.933	<0.001	
	Good	718(53.42)	488(49.90)	230(62.84)			
Hearing	Poor	744(55.36)	568(58.08)	176(48.09)	10.756	0.001	
	Good	600(44.64)	410(41.92)	190(51.91)			
Tumble	No	1,037(77.16)	764(78.12)	273(74.59)	1.881	0.170	
	Yes	307(22.84)	214(21.88)	93(25.41)			
BMI	–	23.48 ± 3.26	23.49 ± 3.17	23.44 ± 3.50	0.252	0.801	
Hypertension	No	860(63.99)	671(68.61)	189(51.64)	33.285	<0.001	
	Yes	484(36.01)	307(31.39)	177(48.36)			
Diabetes	No	959(71.35)	727(74.33)	232(63.39)	15.616	<0.001	
	Yes	385(28.65)	251(25.66)	134(36.61)			
Number of atherosclerotic sites	–	2(2, 4)	2(2, 3)	3(2, 5)	−9.835	<0.001	
LDL-C (mmol/L)	–	2.62 ± 0.89	2.59 ± 0.85	2.72 ± 1.00	−2.412	0.016	
HDL-C (mmol/L)	–	1.3264 ± 0.43	1.3498 ± 0.47	1.26 ± 0.37	3.260	0.001	
TG (mmol/L)	–	1.24(0.94, 1.56)	1.24(0.94, 1.54)	1.27(0.89, 1.61)	−0.156	0.876	
HbAlc (%)	–	5.90(5.40, 6.50)	5.80(5.40, 6.40)	6.10(5.60, 7.00)	−4.837	<0.001	
ADL	Functional impaired	1,048(77.98)	813(83.13)	235(64.21)	55.522	<0.001	
	Normal	296(22.02)	165(16.87)	131(35.79)			Depression
No	1,243(92.49)	922(94.27)	321(87.70)	16.536	<0.001		
Yes	101(7.51)	56(5.73)	45(12.30)				PSQI
Normal	781(58.11)	593(60.63)	188(51.37)	9.398	0.002		
Functional impaired	563(41.89)	385(39.37)	178(48.63)				

BMI, body mass index; LDL-C, low-density lipoprotein; HDL-C, high-density lipoprotein; TG triglycerides; HbAlc, Glycosylated hemoglobin. PSQI, Pittsburgh sleep quality index.

### 3.2 Prevalence and correlates of frailty in older adults with panvascular disease

One thousand three hundred forty-four older adults with panvascular disease were included in the study and the prevalence of frailty was 27.23% (*n* = 366). Comparison between groups showed that there were statistical differences between the frail and non-frail groups in terms of age, gender, education level, Number of atherosclerotic sites, LDL-C, HDL-C and HbAlc (*p* < 0.05). Equal baseline feature distributions between training and validating sets (*p* > 0.05).

### 3.3 LASSO regression and predictive models

**Figure 1:**
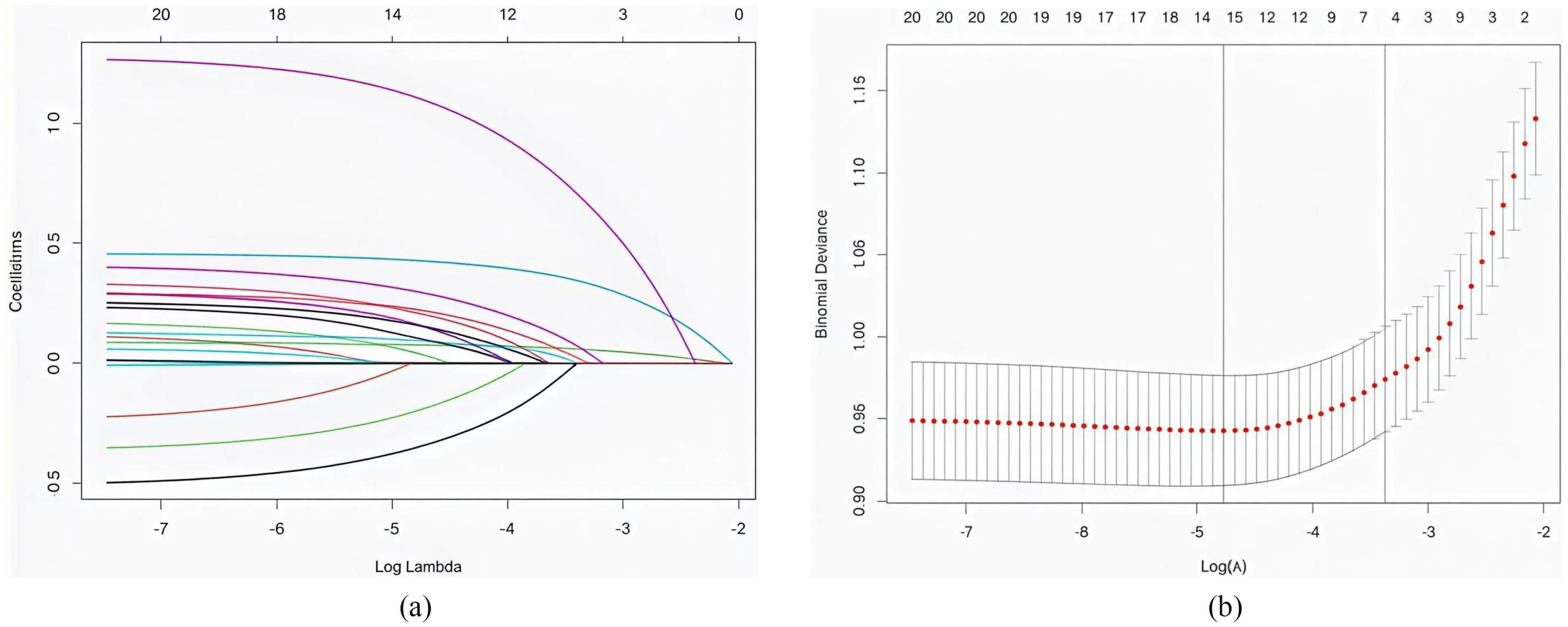
**(a)** The distribution of coefficients was plotted based on the log(lambda) series, with optimal lambda values corresponding to non-zero coefficients. **(b)** Optimal parameters (lambda) for the LASSO model were determined by 10-fold cross-validation with a minimum criterion.

After tenfold cross-validation to optimize the parameters, the LASSO regression model successfully screened 6 key risk factors, including age, number of atherosclerotic lesions, LDL-C, history of hypertension, HbAlc and ADL, when *λ* took the value of 0.034 (Figure 1). After including the above factors in the multivariate logistic regression analysis, it was found that all six indicators were significantly associated with frailty status in older adults with panvascular disease (*p* < 0.05) (Table 2). Collinearity diagnostics for these six variables revealed variance inflation factors (VIF) below 3 for all variables, indicating no significant collinearity interference (Appendix B).

**Table 2:** Multifactorial logistic regression for frailty in older adults with panvascular disease.

Variables	*β*	S. E	Z	*P*	OR (95%CI)
Intercept	−10.93	1.04	−10.48	<0.001	0.00 (0.00 ~ 0.00)
Age	0.09	0.01	7.52	<0.001	1.09 (1.07 ~ 1.11)
Number of atherosclerotic sites	0.48	0.07	7.18	<0.001	1.62 (1.42 ~ 1.85)
LDL-C	0.27	0.09	2.98	0.003	1.31 (1.10 ~ 1.57)
Hypertension					
No					1.00 (Reference)
Yes	0.40	0.17	2.29	0.022	1.49 (1.06 ~ 2.09)
HbAlc	0.15	0.07	2.10	0.035	1.16 (1.01 ~ 1.33)
ADL					
Normal					1.00 (Reference)
Functional impaired	1.20	0.19	6.38	<0.001	3.32 (2.30 ~ 4.80)

### 3.4 Predictive model development

**Figure 2:**
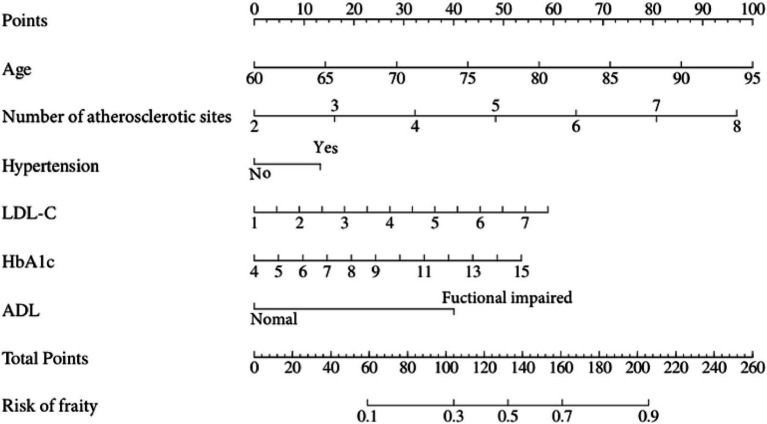
Nomogram for predicting frailty risk in older adults with panvascular disease.

A nomogram that integrates age, atherosclerosis site, LDL-C, hypertension, HbA1c, and ADL was constructed to provide a visual, patient-specific estimate of frailty risk in older adults with panvascular disease (Figure 2).

### 3.5 Predictive model validation

#### 3.5.1 Discrimination

**Figure 3:**
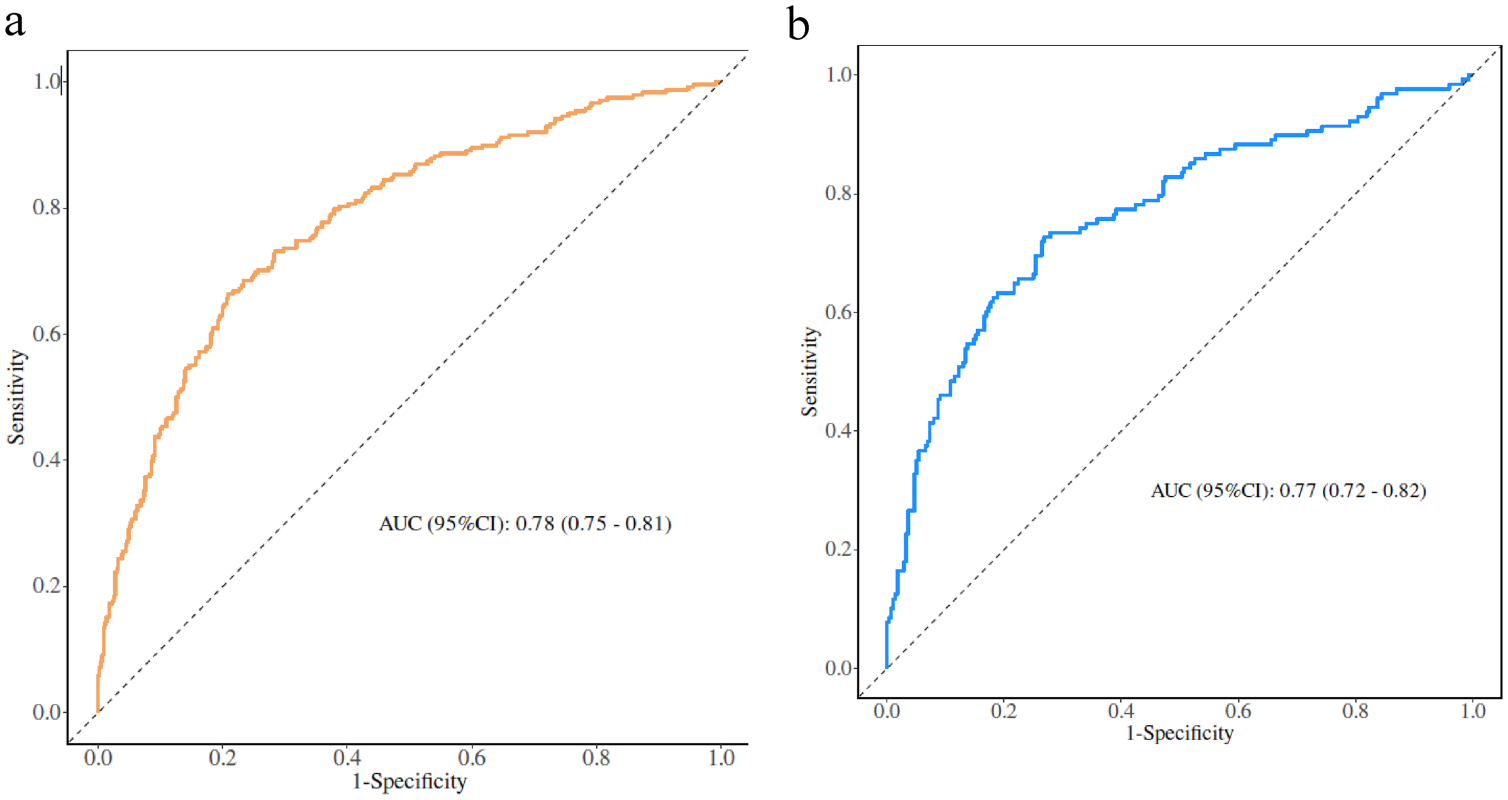
**(a)** Training set. **(b)** Validation set.

The AUC was used to assess the ability of the model to discriminate frailty in older adults with panvascular disease. The AUCs of the training and validation sets were 0.78 (95% CI: 0.75–0.81) and 0.77 (95% CI: 0.72–0.82), the specificity and sensitivity were 0.70, 0.73 and 0.71, 0.74. Through the ROC curve, we determined the optimal cut-off point to maximize both sensitivity and specificity, the optimal cut-off point was 0.285, meaning older adults with panvascular disease whose predicted probability exceeded 0.285 were defined as being at high risk of frailty (Figure 3).

**Figure 4:**
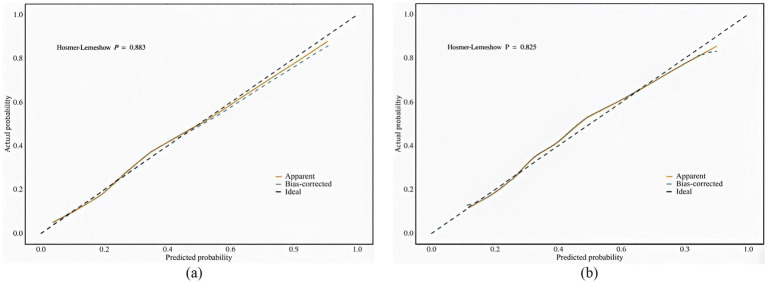
**(a)** Calibration plot for the training dataset. **(b)** Calibration plot for the validation dataset.

Perform internal validation of the constructed model by conducting 1,000 iterations using the bootstrap resampling method. The Hosmer-Lemeshow test confirms that the model is well calibrated on both the training and validation sets (Figure 4). In particular, *χ^2^* = 3.70 (df = 8, *p* = 0.883) for the training set and *χ^2^* = 4.34 (df = 8, *p* = 0.825) for the validation set, with *p* > 0.05, indicating that the model predictions are not significantly different from actual observations. The calibration curve visualization results also show that the model-predicted frailty probabilities are highly consistent with the actual occurrence probabilities, suggesting that the model has good predictive accuracy and reliability.

#### 3.5.2 Evaluation of clinical validity

**Figure 5:**
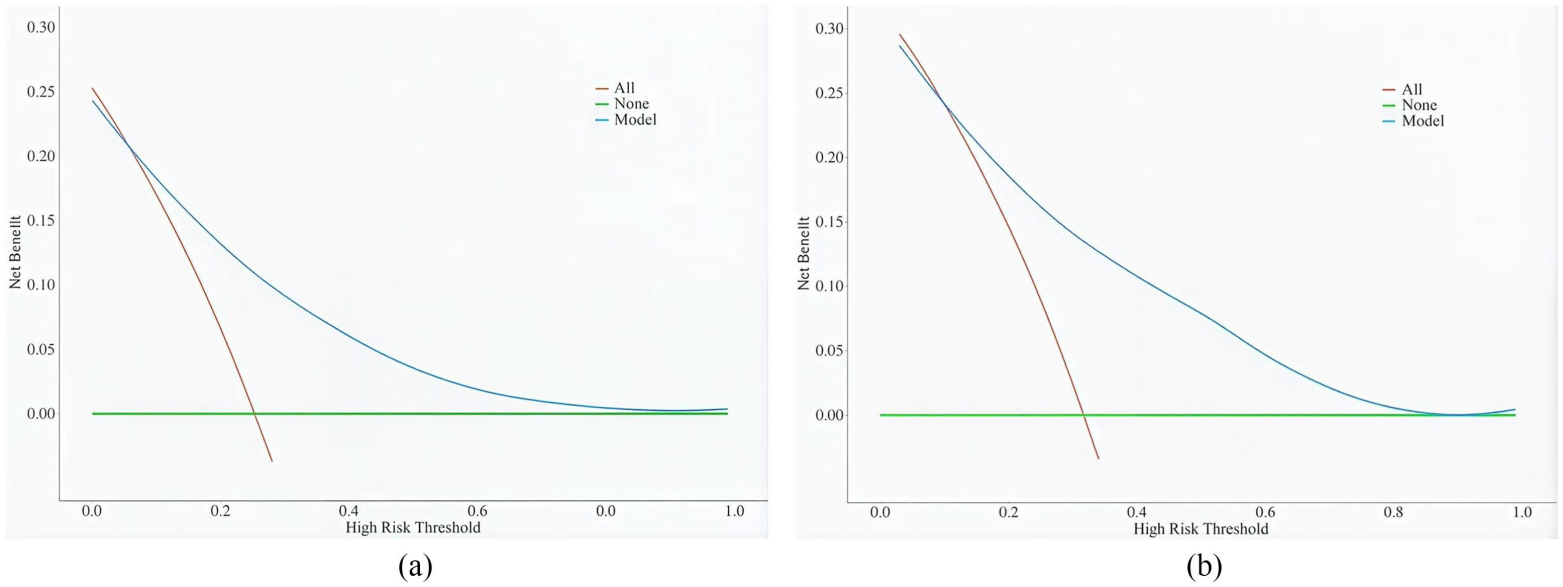
**(a)** DCA curves for the training dataset. **(b)** DCA curves for the validation dataset.

The clinical validity of the model was assessed using DCA (Figure 5). Results show that the model’s net benefit is significantly better than the two extreme internal validation strategies.

## 4 Discussion

In this study, the incidence of frailty in older adults with panvascular disease was 27.23%, which was higher than that in the older population in the community (32, 33), which may be due to the older adults with panvascular disease are more likely to develop frailty-related complications such as metabolic disorders and multi-organ dysfunction. Frailty progression is associated with adverse outcomes (34, 35), and predicting the risk of frailty can improve clinical decision-making and interventions. Therefore, it is of great significance to construct a risk prediction model for pan-vascular weakness in the older adults, identify frailty as early as possible and intervene in time.

This study confirms that age is an independent risk factor for frailty in older adults with panvascular disease, consistent with previous research findings (17, 36). The odds ratio (OR) of 1.09 indicates that for each additional year of age, the risk of frailty increases by 9%. Frailty progressively develops in the older adults, with an average annual incidence rate of 2.9% among those aged 80–84 years (17), representing a 4.58-fold increase compared to the 65–79 age group (33). This mechanism involves age-related declines in multiple systems, including chronic inflammation and cellular senescence.

The number of atherosclerotic sites is a key risk factor for frailty. Studies confirm higher prevalence of grade 3 atherosclerosis in the carotid and lower limb arteries among frail individuals (36). Both share inflammatory and oxidative stress mechanisms, with multi-site lesions causing organ dysfunction through insufficient perfusion across multiple vascular beds. This ultimately manifests as reduced mobility and induces frailty. Clinically, patients with ≥2 affected vascular beds require combined statin therapy with antiplatelet agents (e.g., aspirin), alongside regular vascular ultrasound monitoring to track lesion progression and improve organ perfusion.

Hypertension also constitutes an independent risk factor. Meta-analyses reveal frailty in 23% of hypertensive patients (37), with frail hypertensive individuals aged 65+ exhibiting a threefold increase in all-cause mortality risk (38). Furthermore, systolic blood pressure in frail individuals is 2.8–6.7 mmHg lower than in non-frail individuals (39), potentially due to hypotension impairing major organ perfusion in the older adults and exacerbating ischaemic injury. Patients with panvascular disease who have high HbAlc are at higher risk of developing frailty. Elevated glycated hemoglobin is a marker of hyperglycaemia, and a cross-sectional study conducted in Australia by Hyde et al. (40) found that the prevalence of frailty in those with HbA1c levels ≥6.5% was 70.3%, which may be due to the fact that hyperglycaemia inhibits energy metabolism in skeletal muscle cells, leading to impaired muscle contraction, which in turn causes frailty. Therefore, it is necessary to actively educate patients with panvascular disease about blood glucose management to avoid the adverse effects of hyperglycaemia on the body.

LDL-C levels are important predictors of the progression of panvascular disease. Wang et al. (41) demonstrated that each one-standard-deviation decrease in LDL-C reduces frailty indices by 0.14–0.31%. This may stem from cholesterol deposition in arterial walls causing systemic atherosclerosis, which impairs microcirculation and functional decline in target organs such as the heart, brain, and kidneys, thereby accelerating frailty.

Furthermore, our predictive model suggests that low-level ADL is also a risk factor for panvascular disease frailty (OR = 3.32), meaning frailty risk is 3.32 times higher among ADL-impaired individuals compared to those without impairment. Research indicates that 58.39% of frail patients exhibit ADL impairment (42), a prospective cohort study 4 years ago demonstrated that frailty is significantly associated with a 3.58-fold increased risk of ADL impairment (43). Furthermore, ADL impairment creates a vicious cycle: poor self-care leads to nutritional deficiency, functional decline accompanied by reduced activity induces muscle loss, ultimately exacerbating frailty.

This study has certain limitations: Firstly, the use of convenience sampling for patient selection may introduce selection bias. However, the demographic distribution of our study cohort and the relatively uniform distribution of atherosclerotic lesions across a broad range, coupled with favorable internal validation results, mitigate the impact of selection bias to a certain extent; secondly, Our sample originates solely from a single hospital in the Xi’an region and may not fully represent patient populations in other areas. We shall undertake further multi-center validation in the future. Despite these limitations, the study holds significant value: LASSO regression identified six readily accessible core predictors. Multi-dimensional validation via AUC, Hosmer-Lemeshow tests, and decision curve analysis demonstrated the model’s sound discriminative capability and practicality within the existing sample, providing a reliable framework for subsequent research. Furthermore, addressing the lack of dedicated risk assessment tools for older adults with pan-vascular diseases, the constructed nomogram enables rapid screening of hospitalized patients. This facilitates early intervention for high-risk individuals to mitigate adverse outcomes, while the identified limitations provide clear directions for future optimization.

## 5 Conclusion

This study enabled the assessment of frailty risk in older adults with panvascular disease by constructing and validating a multifactorial model. The model integrates key clinical indicators such as age, number of atherosclerotic sites, history of hypertension, HbA1c and LDL-C. This predictive tool can effectively identify the frail high-risk group among older adults with panvascular disease, providing a simple and effective risk assessment tool for clinical practice, and has important application value for achieving accurate geriatric health management.

## Data Availability

The datasets presented in this article are not readily available because in order to protect the subsequent validation analyses, the dataset of this study will not be disclosed for the time being. Researchers can access the data by making a request to the corresponding author. Requests to access the datasets should be directed to Gao Xia, Gx18291242597@163.com.
