# Supplementary material for: Development and validation of a model to predict the risk of frailty in older adults with panvascular disease
Source: Front Public Health. 2025 Nov 24;13:1631823. doi: 10.3389/fpubh.2025.1631823 (PMC12683717; doi:10.3389/fpubh.2025.1631823)
Supplement: Supplementary file 1 [file Supplementary_file_1.docx]

**Appendix A:**

**Detailed scoring methods for BI, PSQI, GDS-15**

**(1) BI**

The Barthel Index (BI) assessment scale evaluates a patient's ability to perform activities of daily living (ADL). The BI comprises ten items: eating, bathing, grooming, dressing, incontinence, urination, toileting, transfers, mobility, and transfers. Each item is scored according to the level of assistance required by the patient, with a maximum total score of 100 points. A higher score indicates greater independence and lower dependency. In this study, a scale score of 100 points was considered indicative of independent ADL, while scores below 100 points were deemed indicative of non-independent ADL**.**

**(2) PSQI**

Sleep quality was assessed in the survey using the Pittsburgh Sleep Quality Index (PSQI). A total of 19 self-report items were divided into 7 components, including subjective sleep quality, time to sleep onset, sleep duration, sleep efficiency, sleep disturbances, sleep medication use and daytime dysfunction. Each item is scored on a scale of 0 to 3, with a total score ranging from 0 to 21 by adding the scores from the 7 items. A total score greater than 7 indicates poor sleep quality.

**(3)** **GDS-15**

The GDS-15 includes 10 positive items (1 point for "yes" and "no" for "no") and 5 negative items (0 for "yes" and 1 point for "no"). The total score ranges from 0 to 15, with higher scores indicating more severe depressive symptoms and ≥8 indicating the presence of depressive symptoms.
