# Supplementary material for: Development and validation of a model to predict the risk of frailty in older adults with panvascular disease
Source: Front Public Health. 2025 Nov 24;13:1631823. doi: 10.3389/fpubh.2025.1631823 (PMC12683717; doi:10.3389/fpubh.2025.1631823)
Supplement: Supplementary file 2 [file Supplementary_file_2.docx]

Appendix B

Table . Collinearity Diagnosis for Six Variables

| Model | Unstandardized Coefficients | | Standardized Coefficients | t | Sig. | Collinearity Statistics | |
| --- | --- | --- | --- | --- | --- | --- | --- |
|  | B | standard error | Beta |  |  | Tolerance | VIF |
| constant | -1.369 | 0.149 |  | -9.21 | ＜0.01 |  |  |
| Age | 0.014 | 0.002 | 0.243 | 8.252 | ＜0.01 | 0.987 | 1.013 |
| number of atherosclerotic sites | 0.087 | 0.011 | 0.240 | 7.994 | ＜0.01 | 0.947 | 1.055 |
| LDL-C | 0.043 | 0.014 | 0.088 | 2.998 | 0.003 | 0.999 | 1.001 |
| Hypertension | 0.061 | 0.027 | 0.067 | 2.236 | 0.026 | 0.961 | 1.040 |
| HbAlc | 0.024 | 0.011 | 0.063 | 2.110 | 0.035 | 0.968 | 1.034 |
| ADL | 0 | 0.031 | 0.198 | 6.778 | ＜0.01 | 0.998 | 1.002 |
