# Supplementary material for: Development and validation of a model to predict the risk of frailty in older adults with panvascular disease
Source: Front Public Health. 2025 Nov 24;13:1631823. doi: 10.3389/fpubh.2025.1631823 (PMC12683717; doi:10.3389/fpubh.2025.1631823)
Supplement: Supplementary file 3 [file Supplementary_file_3.docx]

Additional file

Title of data: Comparison between variables in the training and validation datasets

**Comparison between variables in the training and validation datasets**

| **Variable** | **Total N = 1344** | **Training set N=940** | **Validation set N =404** | **χ^2^/Z/t** | **P value** |
| --- | --- | --- | --- | --- | --- |
| **Age (year)** | 71(66, 77) | 70(66, 77) | 71(67,77) | -0.449 | 0.654 |
| **Gender** |  |  |  | 3.514 | 0.061 |
| Male | 775(57.66) | 559(59.47) | 218(53.96) |  |  |
| Female | 569(42.34) | 381(40.53) | 186(46.04) |  |  |
| **Marital status** |  |  |  | 0.584 | 0.445 |
| Married | 1061(78.94) | 748(79.57) | 314(77.72) |  |  |
| Unmarried/divorced/widowed | 283(21.06) | 192(220.43) | 90(22.28) |  |  |
| **Education level** |  |  |  | 0.64 | 0.726 |
| Junior high school and below | 748(55.65) | 522(55.53) | 226(55.94) |  |  |
| High school or technical secondary school | 435(32.37) | 309(32.87) | 126(31.19) |  |  |
| College degree or above | 161(11.98) | 109(11.60) | 52(12.87) |  |  |
| **Smoking** |  |  |  | 0.802 | 0.371 |
| No | 1095(81.47) | 760(80.85) | 335(82.92) |  |  |
| Yes | 249(18.53) | 180(19.15) | 69(17.08) |  |  |
| **Alcoholcon sumption** |  |  |  | 0.370 | 0.543 |
| No | 1132(84.23) | 788(83.83) | 344(85.15) |  |  |
| Yes | 212(15.77) | 152(16.17) | 60(14.85) |  |  |
| **Vision** |  |  |  | 0.542 | 0.462 |
| Poor | 626(46.58) | 496(52.77) | 222(54.95) |  |  |
| Good/fair | 718(53.42) | 444(47.23) | 182(45.05) |  |  |
| **Hearing** |  |  |  | 1.622 | 0.203 |
| Poor | 744(55.36) | 531(56.81) | 213(52.72) |  |  |
| Good/fair | 600(44.64) | 409(43.51) | 191(47.28) |  |  |
| **Tumble** |  |  |  | 0.148 | 0.700 |
| No | 1037(77.16) | 728(77.45) | 309(76.49) |  |  |
| Yes | 307(22.84) | 212(22.55) | 95(23.51) |  |  |
| **BMI** | 23.48±3.26 |  |  |  |  |
| **Hypertension** |  |  |  | 2.035 | 0.154 |
| No | 860(63.99) | 613(65.21) | 247(61.14) |  |  |
| Yes | 484(36.01) | 327(34.79) | 157(38.86) |  |  |
| **Diabetes** |  |  |  | 0.009 | 0.924 |
| No | 959(71.35) | 670(71.28) | 289(71.53) |  |  |
| Yes | 385(28.65) | 270(28.72) | 115(28.47) |  |  |
| **Number of atherosclerotic sites** | 2(2, 4) | 2(2, 3) | 2(2, 4) | -0.794 | 0.427 |
| **LDL-C(mmol/L)** | 2.62±0.89 | 2.6203±0.89663 | 2.6241±0.88482 | -0.073 | 0.942 |
| **HDL-C(mmol/L)** | 1.3264±0.432 | 1.3224±0.42696 | 1.3356±0.44677 | -0.515 | 0.606 |
| **TG(mmol/L)** | 1.24(0.94, 1.56) | 1.4271±.79772 | 1.4206±.78832 | 0.137 | 0.891 |
| **HbAlc** | 5.90(5.40, 6.50) | 5.8(5.4, 6.5) | 5.9(5.5, 6.5) | -1.375 | 0.169 |
| **ADL** |  |  |  | 0.084 | 0.771 |
| No | 1048(77.98) | 735(78.19) | 313(77.48) |  |  |
| Yes | 296(22.02) | 205(21.81) | 91(22.52) |  |  |
| **Depression** |  |  |  | 0.021 | 0.885 |
| No | 1243(92.49) | 870(92.55) | 373(92.33) |  |  |
| Yes | 101(7.51) | 70(7.45) | 31(7.67) |  |  |
| **PSQI** |  |  |  | 0.073 | 0.788 |
| No | 781(58.11) | 544(57.87) | 237(58.66) |  |  |
| Yes | 563(41.89) | 396(42.13) | 167(41.34) |  |  |

Description of data: the additional file groups participants according to the training and validation sets, and perform statistical analysis of the basic characteristics of the two groups, with the results presented as a table.
